# Classical Swine Fever in China-An Update Minireview

**DOI:** 10.3389/fvets.2019.00187

**Published:** 2019-06-13

**Authors:** Bin Zhou

**Affiliations:** MOE Joint International Research Laboratory of Animal Health and Food Safety, College of Veterinary Medicine, Nanjing Agricultural University, Nanjing, China

**Keywords:** classical swine fever, CSF virus, China, epidemiology, control strategy

## Abstract

Classical swine fever (CSF) remains one of the most economically important viral diseases of domestic pigs and wild boar worldwide. The causative agent is CSF virus, it is highly contagious, with high morbidity and mortality rates; as such, it is an OIE-listed disease. Owing to a nationwide policy of vaccinations of pigs, CSF is well-controlled in China, with large-scale outbreaks rarely seen. Sporadic outbreaks are however still reported every year. In order to cope with future crises and to eradicate CSF, China should strengthen and support biosecurity measures such as the timely reporting of suspected disease, technologies for reliable diagnoses, culling infected herds, and tracing possible contacts, as well as continued vaccination and support of research into drug and genetic therapies. This mini-review summarizes the epidemiology of and control strategies for CSF in China.

## Introduction

Classical swine fever virus (CSFV), is a *Pestivirus* in the Flaviviridae family. It is highly contagious and causes disease that can be acute (i.e., transient or lethal) or chronic. Disease progression is dependent on a number of factors, such as strain virulence, host factors, and secondary pathologies. Typically though the acute disease is characterized by high fever, inappetence, and general weakness followed by neurological deterioration, petechial hemorrhages of the skin, and splenic infarction (1, 2). These acute CSFV infections result in high morbidity and mortality rates can be as high as 100%. Subclinical signs such as intermittent fever and inappetence can be seen in chronically infected pigs, and although not life threatening, morbidity is still high (3, 4).

Because of its worldwide distribution and its immense economic impact on the porcine industry globally (5–7), CSF is reportable to the World Organization for Animal Health (OIE) (8). China has also classified CSF as a class A animal infectious disease (9), and according to the National Medium-Term and Long-Term Animal Disease Control Program issued in 2012, CSF, along with other the major animal diseases (Newcastle Disease, Foot-and-Mouth Disease, Highly Pathogenic Avian Influenza), is deemed “most important” and has priority status in disease prevention and control programs (10).

Domestic pigs and wild boars are the known reservoirs for CSFV (11). Since its initial identification in 1833 in the United States, CSFV spread worldwide (12). In recent decades, many countries have implemented strategies for surveillance and control (13). Essential elements of any effective strategy include early diagnosis, culling of infected pigs, formulation and implementation of appropriate veterinary regulations, environmental rehabilitation, as well as prophylactic measures. Where well-implemented these policies have proven remarkably successful in controlling CSF (14). Canada successfully eliminated CSF in 1963, followed by the USA in 1976 and Mexico in 2018 (oie.int); recent data from the World Organization for Animal Health released show that there are now approximately 34 CSFV-free countries (www.oie.int). In areas with dense wild boar populations CSF tends to become endemic whereas it is often self-limiting in small, less dense populations. There has however been a disturbing trend of recurrence in some countries that had declared CSF eliminated (France, the Netherlands, Germany, and Belgium) (6, 15). Parts of Asia and South America have also seen an uptick in cases, of note are the recent reports from Japan of a few documented cases (16).

## Epidemiological Characteristics

### Current Epidemiology

China has the largest pig breeding industry in the world, accounting for more than half of global production along with ~40 million sows and 7 billion fattening pigs (17). According to the Veterinary Bulletin of China, there were 475, 268, 115, 28, 28 cases documented in 2010, 2011, 2012, 2013, and 2014, respectively. There were only 21 cases documented in 2017. The results showed that CSF outbreaks in China has been decreasing over time in recent years (18, 19). As encouraging as this data is, challenges remain for China in the effort to eradicate CSF (20, 21). As the epidemic outbreaks of past years have largely been replaced by sporadic outbreaks, and the virulence of wild type CSFV has decreased, the course of disease has shifted from acute and sub-acute to a chronic form. In addition, there are well documented reports that CSFV may spill over directly or indirectly from wild boar to domestic pigs (6). It was proven that 60% of 92 cases were caused by direct or indirect contact with wild boar (22) in Germany. Remarkably, Japan has reported many cases of CSF in wild boars last September (16). However, there are few cases of virus transmission between wild boar and domestic pig in China (23).

A major challenge facing China is preventing the sporadic outbreaks of CSF on the smaller and medium pig farms (24, 25). Large-scale pig farms have very high immunization rates, as all pigs (boars, sows, and growing and fattening pigs) are immunized, but small and medium-sized farms are not as well-supported and face problems with immunization, these include: (1) immune tolerant gilts are not eliminated before entering the population, (2) immunization procedures are not standardized and do not follow the curve of maternal antibody, therefore, piglets may not receive sufficient immunization, (3) antibody titer is not monitored annually. In this case, even as the population receives cohort immunization, the immune effect is not ideal (26, 27). Clearly better prevention and control measures, with the support from the Veterinary Bureau, are needed to eradicate CSF in China.

### Mixed Infections

Co-infection by CSFV and other pathogens complicates diagnosis, treatment, and prevention protocols; as a result morbidity and mortality rates can be quite high. In China, commonly found coinfections with CSFV are porcine reproductive and respiratory syndrome virus (PRRSV), pseudorabies virus (PRV), porcine circovirus type 2 (PCV2), swine influenza viruses (SIV), and often secondary infection such as *Haemophilus parasuis*, swine *pasteurellosis, Streptococcosis*, swine enzootic pneumonia, paratyphoid, *colibacillosis, toxoplasmosis*, and *eosporophilosis* (28). Some cases have been currently reported that PRRSV and CSFV coinfections are common in Chinese pig populations (29, 30). This combination of pathogens is particularly costly to the Chinese pig industry, because PRRSV is immunosuppressive it seriously inhibits the immune response to the CSF vaccine. Further reports have shown that two other *Pestiviruses*, BVDV and BDV, strongly inhibit the immune response of vaccine against CSFV (31, 32). Based on a coinfection model for PCV2 and CSFV, bioinformatic analyses indicated that mitochondrial dysfunction, nuclear factor erythroid 2-related factor 2 (Nrf2)-mediated oxidative stress response and apoptosis signaling pathways might be the specific targets during PCV2-CSFV coinfection (33). These cases highlight the complexity of CSF control in China.

## Geographical Distribution of Genotypes

China and surrounding countries, especially countries of Southeast Asia, have long been the epidemic areas (34). Broadly speaking, molecular epidemiology seeks understand how the interaction of genetic traits and environmental factors result in disease (35). CSFV is a positive single-stranded RNA virus, with a genome approximately 12.3 kb; it comprises a single open reading frame (ORF) that is translated into a single polyprotein composed of 3,898 amino acids. The coding region is flanked by two non-coding regions at both ends (5′ UTR and 3′ UTR) (36, 37). Phylogenic typing has been based on partial sequences of 5′-UTR, E2, and the polymerase gene 5B (NS5B). CSFV isolates worldwide are divided into three genotypes and 11 subgenotypes (1.1, 1.2, 1.3,1.4, 2.1, 2.2, 2.3, 3.1, 3.2, 3.3, and 3.4) (38–41); subgenotype 2.1 is further divided into sub-subgenotypes 2.1a, 2.1b, 2.1c, and 2.1d (42, 43). While globally genotype 2 has been the most prevalent in the last few decades (44–46), all isolates from the Americas belong to genotype 1. The Cuban isolates are clustered in subgroup 1.2, the isolates from Honduras and Guatemala are clustered in subgroup 1.3, and the isolates from Argentina, Brazil, Colombia, and Mexico generated four poorly resolved clusters in subgroup 1.1. However, a present report demonstrated that the Cuban isolates are more divergent from other so far known CSFV subgenotype 1 isolates and form a novel separate subgenotype that is proposed to be designated subgenotype 1.4 (47, 48). Apart from the CSF outbreak in South Africa in 2005 and in Israel in 2009, which were caused by subtype 2.1, very little is known about CSFV in Africa and the Middle East (49). The reports in India show that there is a mixed population of subgenotypes 1.1, 2.1, and 2.2 co-circulating; historically subtype 1.1 was dominant (50–52). The global distribution of subtypes is shown in Table 1.

**Table 1:** Global distribution of CSFV subgenotypes.

**Genotypes of CSFV**	**Countries**
1.1	Argentina, Brazil, Colombia, Mexico, Italy, Russia, India, China
1.3	Honduras, Guatemala
1.4	Cuba
2.1	South Africa, Germany, The Netherlands, Italy, Spain, Belgium, Croatia, Lithuania, Israel, India, Korea, China, Taiwan, Laos, Mongolia, Indonesia, Vietnam
2.2	Germany, Italy, Czech Republic, Former Yugoslav Republic of Macedonia, India, Nepal, Laos, China
2.3	Italy, Croatia, France, Romania, Bulgaria, Serbia, Slovakia, Czech Republic, Russia, China
3.2	Korea
3.3	Thailand
3.4	Taiwan, Japan

*The data in this table have been published in the past 20 years*.

There is a high degree of variation among the prevalent strains of CSFV in China. In the 1990s, the main epidemic strains of CSFV in mainland of China belonged to subtypes 1.1, 2.1, 2.2, and 2.3 (53). Subtypes of 2.1, 2.2, and 2.3 were predominant in provinces with the most developed pig industry, such as Beijing, Hubei, Jilin, Sichuan, Fujian, Henan, Guangxi and Inner Mongolia. However, these 4 subgenotypes are endemic in Guangdong Province. Since the beginning of the 21th century subgenotypes 2.2 and 2.3 have become less dominant. Presently, 2.1 is the prevalent subgenotype in mainland China (41–45), and 2.1b has become the dominant epidemic subgenotype (54, 55), with 2.1c becoming dominant in southern China (42) and in Taiwan subgenotype 3.4, the dominant subgenotype before 1996, has gradually been replaced by 2.1 (41). Jiang et al. (56) analyzed sequences of 8 epidemic strains isolated in Hunan in 2011–2012, and found that 5 isolates formed a single evolutionary branch of the 2.1 subgenotype along with the isolates from Guangdong and Guangxi. Isolates forming this new branch are designated subgenotype 2.1c. Subgenotype 2.1c is also distributed in Thailand and Laos. Tu et al. (53) showed that subgenotype 2.1c appeared in Guangxi as early as 1998 and Peng et al. (57) further divided 15 isolates of subtype 2.1 from Guangdong in 2011 into three subgenotypes of 2.1b, 2.1c, and 2.1d, while the prevalence of 2.1c and 2.1d was the first reported in Guangdong (32, 58). The distribution of genetic diversity is probably related to the transportation of pigs and the level of development of the pig industries.

In order to further understand the genetic diversity of CSFV in China, 39 isolates from Guangdong and Guangxi from 2004 to 2012 were sequenced and analyzed. Based on partial E2 gene fragment (190 nt) and full-length E2 gene sequence (1119 nt), phylogenetic analysis showed that the currently prevalent subgenotype 2.1 can be further divided into 10 sub-subgenotypes (2.1a~2.1j), and the isolates previously identified by Peng *et al* as 2.1d are now reclassified into subgenotype 2.1g (57). According to temporal and spatial distribution characteristics, the currently most prevalent subgenotype is 2.1b, the second prevalent subgenotypes are 2.1d and 2.1 g, and the silent subgenotypes are 2.1a, 2.1e, and 2.1f (59).

In summary, all four subgenotypes existed before 2008; 2.1 was the most predominant, followed by 1.1, 2.2, and 2.3 which were geographically scattered. Under pressure of the C strain vaccine (1.1), the prevalence of subgenotype 1.1 gradually decreased, and subgenotypes 2.2 and 2.3 gradually withdrew from the epidemic areas, leaving subgenotype 2.1, which is the most phylogenetically distant from the vaccine strain, the dominant CSFV strain in China (60). The epidemic strains in China are genetically diverse, the most prevalent genotype 2 strains are related to those from Europe, possibly originating from the same viral ancestor. We speculate that it may be due to the long-term introduction of pig breeds from EU countries. Although, the epidemic strain of genotype 3 has not been reported in China, it is necessary to maintain surveillance to prevent its introduction from areas surrounding China, such as South Korea (61), Taiwan, and Japan (16).

## Evolution of Variants and Vaccine Protection

In the more than 60 years since the safe and effective attenuated vaccine was developed in 1954 and used in China (62), CSF has been effectively controlled but not eradicated. In recent years, CSF outbreaks have tended to occur sporadically. Since 2015, the abortions, stillbirths, and diarrhea have increased gradually. Whether these conditions are related to the changes of subgenotypes has not been effectively verified. However, it is certain that there are genetic differences among different subgenotypes (63).

E2 (gp55), the envelope glycoprotein is where most of the antigenic epitopes of CSFV are concentrated, it is highly immunogenic and induces neutralizing antibodies (64). The mutation rate of E2 is between 3 and 25%, it is one of the regions with the greatest mutation rates (65, 66). The percent homology between full-length E2 genes of CSFV subgenotypes is shown in Table 2. Note that the percent homology between subgenotype 2.1 and 1.1 (the vaccine strain) is the lowest, suggesting the reason why subgenotype 2.1 is the main epidemic in China (56).

**Table 2:** Percent nucleotide homology of full-length E2 between CSFV genotypes (56).

**Genotype**	**2.1c(%)**	**2.1a(%)**	**2.1b(%)**	**2.2(%)**	**2.3(%)**	**1.1(%)**	**1.2(%)**	**3.4(%)**
2.1c	94.8–100	90.2–94.9	89.9–93.8	87.3–90.1	84.5–85.4	80.8–84.5	81.9–93.0	81.6–83.1
2.1a		94.9–100	91.1–95.7	87.3–91.3	86.5–89.6	81.7–85.7	83.3–85.0	81.4–83.7
2.1b			93.3–100	87.1–89.0	86.4–89.0	81.4–84.4	81.7–83.7	81.2–82.5
2.2				93.7–100	86.5–90.7	81.6–85.0	82.9–85.5	82.3–84.0
2.3					94.6–100	80.7–85.1	82.0–83.6	80.5–81.9
1.1						91.8–100	88.2–93.1	81.6–85.7
1.2							94.2–100	84.4–84.8
3.4								98.3–100

Many studies have shown that vaccination has exerted an influence on the evolution of classical swine fever virus (67). In recent years, a number of immune escape mutant strains, those that are not neutralized by polyclonal antibodies against C strain, have been identified (68). Therefore, we asked whether genotype 2.1 has characteristics of these immune escape mutant strains, while genotype 2.2 and 2.3 gradually disappear under vaccine-induced immune pressure. Results of a cross neutralization test show that the neutralizing ability of the immune pig serum against the C strain is not significantly different from that of the 2.1 major subtype strains that were prevalent in the late 20th century, indicating that the antigenicity of genotype 2.1 has not changed significantly over time, but that its neutralization ability is lower than that of genotype 2.2 and 2.3. This suggests that genotype 2.1 may survive more easily in the natural immune environment, though of course, this speculation needs further study.

To further investigate viral gene variation, we compared the amino acid and nucleotide sequences of the E2 gene of the C strain vaccine with those of epidemic strains isolated from different regions in China from 2010 to 2015. We found 79.4–99.0% nucleotide homology and 78.0–97.9% amino acid homology (Table 3).

**Table 3:** Comparison of the homology of the isolates from different regions and laboratories from 2010 to 2015, compared with that of C strain.

**Background**			**Between the isolates**		**Compared with C strain vaccine**	
**References**	**Year**	**Strains**	**Nucleotide%**	**Amino acid%**	**Nucleotide%**	**Amino acid %**
Fu et al. (69)	2010	7	96.3–99.3	95.6%−100	80.6–81.7	78.0–80.2
Zhu et al. (70)	2011	53	90.15–100	/	79.4–83.1	81.5–85.4
Wang et al. (71)	2012	26	87.1–100.0	/	79.9–91.4	/
Huang et al. (72)	2012–2014	30	81.0–100	85.4–100	81.1–99.0	85.8–97.9
Guo et al. (73)	2012–2014	2	98.7	98.7	83.1–83.6	/
Feng et al. (74)	2015	14	/	/	81.1–82.4	88.2–89.8

In general, the homology of nucleotide and amino acid sequences between the isolates and C strain is about 80%, except for some isolates that were very similar to the C strain. Given that the % homology of E2 and other major antigenic proteins between the isolates and the C strain is quite different, does this indicate that the vaccine is failing to provide effective immune protection for pigs? Wang et al. (75) studied the immuno-protective effect of the C strain vaccine against 9 genotypically different strains epidemic in China that present with different clinical pathogenicities. The results showed that the C strain vaccine did produce protection against the tested strains, subgenotypes 1.1, 2.1, and 2.2. Importantly the immunized pigs that were challenged with the test strains did not shed virus. These results provide a scientific basis for the continued use of C strain vaccine in China, but in order to eradicate CSF, it will not be enough. It is not possible to distinguish between vaccinated and naturally infected animals, therefore, the new labeled vaccine will play an important role (76, 77). Up to very recently, only E2 subunit marker vaccines were available on the market (20, 21, 78). In 2014, a new live attenuated marker vaccine CP7_E2alf was licensed by the European Medicines Agency. The resulting data from Friedrich- Loeffler-Institut showed that “CP7_E2alf” is a new instrument in the tool-box of CSF control and can be used to revisit emergency vaccination scenarios (79). Although the vaccines are currently sufficient to provide effective immune protection, they are not omnipotent. In order to cope with future crises, China should strengthen biosafety through continued vaccination and developing alternative methodologies in order to realize the eradication of CSF.

## Eradication Strategies

There are two strategies for CSF control in the world: preventive immunization and comprehensive culling. For most countries that have no endemic CSF, such as the United States, Canada, Brazil, Chile, South Africa, and the EU countries, culling is used to control CSF. In China, large-scale culling is not feasible, for the present prophylactic vaccination is the best way to reduce the CSF disease burden. The C strain vaccine is widely used in China, but in addition to the problem of being unable to distinguish naturally infected pigs from immunized pigs, use of the C strain vaccine poses other practical problems such as immunization optimization, immunosuppression, vaccine quality, and of course availability and compliance. For example, antibody levels of sows may be above 90%, but the antibody titers of nursery pigs is uneven. Chinese scientists have been working hard to develop new gene-labeled and E2 subunit vaccines for many years and these will be powerful tools for CSF eradication (80, 81). A recombinant E2 subunit vaccine, Rb-03 strain, was developed by Xinjiang Tiankang Animal Husbandry Biotechnology Co., Ltd. in 2016. After vaccination, with this engineered strain, pigs were challenged with CSFV Shimen strain. Challenged pigs did not show clinical signs of CSF and cleared the virus quickly. If such vaccines can produce reliable clinical protection, Chinese pigs may be no longer be diseased by the Shimen strain. The protective efficacy of the subunit vaccine was not different from that of the C strain. The latest unpublished data showed that E2 subunit vaccine can induce 100% protection against subgenotypes 2.1b, 2.1c, 2.1h, and 2.2.

The biosecurity levels in large-scale pig farms are constantly improving as the Chinese government gives more priority to CSF eradication policies. Listed below are some specific conditions that need to be pursued if the goal of CSF eradication by the end of 2020 is to be met. The conditions are: (1) Cooperative prevention and control. In addition to monitoring and documenting CSFV infection rates and antibody levels, we should also closely monitor the other important swine diseases that are often coinfections with CSFV (such as PRRS, PCAD, PR, etc.) (29, 82, 83); (2) Technical support. An eradication program needs skilled veterinarians, up-to-date diagnostic and monitoring technologies; (3) High quality vaccines must be widely available; (4) Maintain, or pursue where needed, high quality biosafety. Twice yearly etiological investigations should be conducted and where possible, pigs testing positive for pathogens should be culled.

The development of CSFV antigen and antibody detection technologies are important for the prevention and control of CSF. For example, epidemiological investigation and real-time monitoring of antibody levels in immunized pigs are indispensable steps in the process of eradication. Currently there are many diagnostic methods, among which the diagnosis of clinical symptoms is the most direct. But even professional veterinarians are prone to misjudgment in the diagnosis of clinical symptoms and pathological changes. Therefore, to get more reliable results, immunology and molecular biology methods are commonly used to determine levels of CSFV infection as follows (84). The most common immunological detection methods in China are immunofluorescence technology (IFA), virus neutralization tests (VNT), immune colloidal gold technology (GICT), and enzyme linked immunosorbent assay (ELISA), which is the most widely used. Commercial test kits provide rapid reliable detection, greatly improving detection efficiency by allowing for early diagnosis and efficient immune surveillance. It is however still impossible to distinguish between vaccinated and infected animals, and further research is needed. Xu et al. (85) used eukaryotic expression methods to express CSFV E2 protein then purify it from an inclusion. They then developed an indirect ELISA, thereby laying a solid foundation for the development of a diagnostic kit. In recent years, more and more research to detect antibodies and pathogens in the oral fluids of swine has been reported. With the rapid development of molecular biology technologies, their role in the diagnosis of animal diseases have become prominent. Presently, the most widely used CSFV nucleic acid detection technologies, RT-PCR, RT-nested PCR, RT-nested PCR based restriction fragment length polymorphism (RFLP), real-time RT-PCR, and RT-LAMP, have been developed in China to detect CSFV and/or differentiate wild-type CSFV and C-strain. Due to co-infections of CSFV with other viruses, several multiplex PCR assays have been developed in China, allowing simultaneous detection of CSFV and other porcine viruses.

Depending on vaccination alone though, may not be sufficient to eradicate CSF and the development supplemental antiviral strategies are needed. Anti-CSFV therapies such as capsid-targeted virus inactivation (86), RNA-hydrolyzing recombinant antibody (87), RNA interference (88), Imidazo[4,5-c]pyridines (89), and uridine derivatives of 2-deoxy sugars (90) have been reported but their clinical effect and practical application for CSF control needs further study and development. Our lab has found porcine Mx1 has anti-CSFV activity (91) and continue to dissect the mechanism of poMx1 against CSFV (92). Our findings will provided significant information for the potential development of a novel antiviral therapy. In addition, our research clarified the pathway of CSFV internalization (93, 94), which will promote our current understanding of pestivirus cellular entry pathways and provide novel targets for antiviral drug development. Finally, anti-CSFV transgenic pigs have been produced by somatic nuclear transfer and *in vitro* and *in vivo* viral challenge assays have demonstrated that replication of CSFV and CSFV-associated pathologies and mortality in these pigs is effectively limited (95), and a recent report that transgenic pigs refractory to CSFV have been successfully developed using a CRISPR/Cas9-mediated knock-in strategy, offers exciting promise (96). Interestingly, we know that the host factor JIV can promote viral replication (97, 98). If the researchers use the CRISPR/Cas9 technology to knock out the JIV gene and breed another pig that is resistant to CSFV, it is possible in the future.

## Author Contributions

The author confirms being the sole contributor of this work and has approved it for publication.

### Conflict of Interest Statement

The author declares that the research was conducted in the absence of any commercial or financial relationships that could be construed as a potential conflict of interest.
